# Factors associated with choice of high lethality methods in suicide attempters: a cross-sectional study

**DOI:** 10.1186/1752-4458-8-43

**Published:** 2014-11-18

**Authors:** Sang Hoon Oh, Kyoung Uk Lee, Soo Hyun Kim, Kyu Nam Park, Young Min Kim, Han Joon Kim

**Affiliations:** Department of Emergency Medicine, Seoul St. Mary’s Hospital, College of Medicine, The Catholic University of Korea, 222 Banpo-daero, Seocho-Gu, Seoul, 137-701 Republic of Korea; Department of Psychiatry, College of Medicine, The Catholic University of Korea, Seoul, Republic of Korea

**Keywords:** Attempted suicide, Poisoning, Methods

## Abstract

**Background:**

Most attempted suicides have a low lethality, but hanging, drowning, and jumping from a great height have a high risk of completed suicide. The aim of this study was to assess the sociodemographic profiles of patients who attempted suicide using high lethality methods relative to all other methods of attempted suicide.

**Methods:**

We retrospectively investigated all attempted suicides treated at a tertiary university hospital in Seoul between January 2008 and February 2012. The following variables were considered: the patients’ attempted suicide methods, age, sex, history of attempted suicides, previous psychiatric history, occupation, and living conditions. The suicide methods were categorized into two groups: high lethality (e.g., hanging, falling, and drowning) and low lethality methods (e.g., self-poisoning and cutting). We investigated risk factors related to the choice of high lethality methods.

**Results:**

A total of 560 patients were enrolled in this study. Deliberate self-poisoning was the most common method of attempted suicide (61.6%), followed by cutting (22.5%), hanging (10.4%), falling (4.1%), and drowning (1.4%). In logistic regression analyses, odds ratios for the choice of high lethality methods were 1.02 (95% CI = 1.01 to 1.03, p < .01), 7.22 (95% CI = 3.06 to 17.04, P < .01), and 0.59 (95% CI = 0.35 to 0.99, p = .04) for age, previous attempted suicide with a high lethality method, and alcohol co-ingestion, respectively.

**Conclusions:**

Our findings indicated that age and past attempted suicide using a high lethality method are associated with the use of high lethality methods for attempting suicide.

## Background

Suicide rates vary geographically, but suicide is one of the major causes of mortality worldwide and an important public health problem [1–3]. In Korea, suicide is currently the most serious and urgent public health issue. Korea has the highest suicide rate among member countries of the Organization for Economic Cooperation and Development (OECD). In 2011, Statistics Korea reported a suicide rate of 31.7 per 100,000 people, which is 2.6 times greater than the OECD average and represents a twofold increase in suicides over the last decade [4]. Suicide is the fourth leading cause of death following cancer, stroke, and cardiovascular disease [4]. Park and Lester’s study on suicide in Korea using the 2005 suicide data reveals that suicide rates were higher in rural areas than in urban areas [5]. Those in rural areas more often used pesticides and chemicals as a method of suicide, and there was a greater proportion of men and the elderly, both groups at higher risk for suicide in Korea. And Kim et al. suggest that the current suicide epidemic in Korea has social origins such as lower education, rural residence, and area deprivation [6].

Most attempted suicides involve self-poisoning or cutting, which are typically classified as low lethality methods. On the other hand, hanging, drowning, the use of firearms or explosives, and jumping from a great height are less common but are classified as high lethality methods and have a high risk of completed suicide [7, 8]. Although the factors influencing an individual’s suicide method may include sociocultural acceptability and media portrayals of suicide [9, 10], little is known about which factors are related to an individual’s suicide method. Understanding the characteristics of attempted suicides using high lethality methods will help in designing suicide prevention strategies.

In this study, we evaluated the association between the sociodemographic characteristics of individuals who attempted suicide and their choice of suicide methods, with the aim of identifying risk factors for choosing high lethality methods.

## Methods

We retrospectively reviewed the medical records of patients who had visited the emergency department (ED) of a tertiary university hospital for treatment following an attempted suicide between January 2008 and February 2012. The present study was approved by the Institutional Review Board of the Catholic University of Korea, Seoul Saint Mary’s Hospital.

In Korea, patient information on the cause of ED admission is immediately reported to the National Emergency Department Information System (NEDIS) records. If the cause of admission was a deliberate self-harm, information on the method used for each attempted suicide was recorded as either poisoning, cutting or piercing, suffocation, hanging or choking, drowning or near drowning, using fire or heat, falling or jumping from a great height, other method, or unknown. All patients who had visited our ED after attempted suicides were included in the NEDIS records. Two emergency physicians independently reviewed medical records of these patients.

The variables included suicide methods, sociodemographic characteristics, and hospital discharge outcomes. Suicide methods were dichotomized into low lethality and high lethality methods. Low lethality methods are the most common methods of parasuicide and are sometimes known as soft methods. Such methods include self-poisoning and self-harm such as cutting or piercing [8]. High lethality methods are followed by more serious injuries, for example, hanging, drowning, falling, or using fire or heat. Sociodemographic variables included the number of attempted suicides, family status, occupation, alcohol use, and past psychiatric history. In addition, all medical records, including the discharge records, were reviewed to determine whether the suicide was completed (i.e., the patient died) prior to hospital discharge.

The distribution of patient characteristics is presented as either a percentage or the mean (± standard deviation). To compare the distribution of the characteristics between the two groups, we used Student’s t-test for continuous variables and the chi-squared test for categorical variables. Sociodemographic factors related to high lethality methods were evaluated with a multivariate logistic regression analysis; odds ratios (OR) and 95% confidence intervals (CI) were estimated in the logistic regression model. In addition, compared with patients using self-poisoning, the ORs of other methods for completed suicides were evaluated using a multivariate logistic regression analysis. All statistical analyses were performed using SPSS 16 (SPSS, Chicago, IL), and differences with a p-value < .05 were considered statistically significant.

## Results

**Figure 1:**
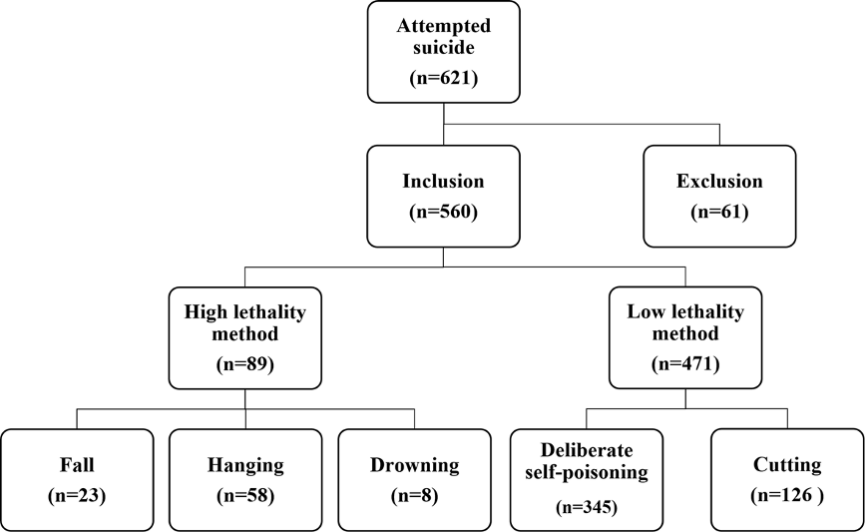
**Flow chart of study inclusion.**

There were 621 cases of attempted suicides during the study period, which constituted 0.27% of total ED visits during this period. For 61 patients with self-cutting to the wrist, extensive records (including assessments by the psychiatrists) were incomplete. These patients without complete medical records were excluded from this study. As a result, a total of 560 patients were enrolled in this study (Figure 1). Deliberate self-poisoning was the most commonly chosen method for attempted suicide (61.6%), followed by cutting or piercing (22.5%), hanging (10.4%), falling (4.1%), and drowning (1.4%). Table 1 shows the baseline sociodemographic characteristics of study cohort. The mean age of the 560 patients was 37.6 (±17.2) years and ranged from 12 to 94 years; 66 (11.8%) were teenagers, and 54 (9.6%) were aged 65 years or older. Of the patients, 267 (47.7%) were male and 293 (52.3%) were female. In total, 167 (29.8%) patients presented with repeated attempted suicides. Of these, 24 (14.4%) patients had made a previous attempt using a high lethality method. 184 (32.9%) were not in the labor force. In addition, 133 patients (23.8%) were living alone. There was co-ingestion of alcohol in 201 (35.9%) attempted suicides and a total of 221 patients (39.5%) reported previous psychiatric treatment.

**Table 1:** **Baseline sociodemographic characteristics and discharge outcomes of study cohort and comparison between the high lethality methods and low lethality methods groups**

	Total (n = 560)	High lethality methods (n = 89)	Low lethality methods (n = 471)	***p***
Age	37.6 ± 17.2	42.1 ± 19.3	36.7 ± 16.6	.02
≥ 65	54 (9.6)	15 (16.9)	39 (8.3)	.01
Male	267 (47.7)	39 (43.8)	228 (48.4)	.25
Reattempt	167 (29.8)	20 (22.5)	147 (31.2)	.06
Critical method history	24 (5.2)	12 (13.5)	12 (2.5)	< .01
Unemployed	184 (32.9)	39 (43.8)	145 (35.4)	.09
No family	133 (23.8)	16 (18.0)	117 (24.8)	.10
Alcohol coingestion	201 (35.9)	23 (25.8)	178 (37.8)	.02
Psychiatric history	221 (39.5)	37 (41.6)	184 (39.1)	.31
Mood disorder	182 (32.5)	31 (34.8)	151 (32.1)	.35
Schizophrenia	20 (3.6)	5 (5.6)	15 (3.2)	.20
Other	21 (3.8)	1 (1.1)	20 (4.2)	.13
Death prior to discharge	50 (8.9)	39 (43.8)	11 (2.3)	< .01

Parenthesis indicates percentage.

We categorized patients into a high lethality methods group (n = 89) and a low lethality methods group (n = 471) and examined the sociodemographic characteristics of each. The average ages of patients in the high lethality methods group and the low lethality methods group were 42.1 (±19.3) and 36.7 (±16.6) years, respectively (p = .02); older patients (≥65 years) were more likely to choose high lethality methods (p = .01). There were no significant differences between the two groups regarding sex, number of previous attempted suicides, occupation, family condition, and history of past psychiatric disorders. Previous attempted suicides using high lethality methods were significantly higher in current attempted suicides using high lethality methods (p < .01). On the other hand, alcohol co-ingestion (p = .02) was significantly higher with attempted suicides using low lethality methods (Table 1).

We performed a multivariate logistic regression analysis to investigate the relationship between high lethality methods of attempted suicide with factors including age, previous attempted suicide using high lethality methods, and alcohol co-ingestion (Table 2). The ORs for high lethality methods were 1.02 (95% CI = 1.01 to 1.32, p < .01), 7.22 (95% CI = 3.06 to 17.04, p < .01), and 0.59 (95% CI = 0.35 to 0.99, p = .04) for age, previous attempted suicide using high lethality method, and alcohol ingestion, respectively.

**Figure 2:**
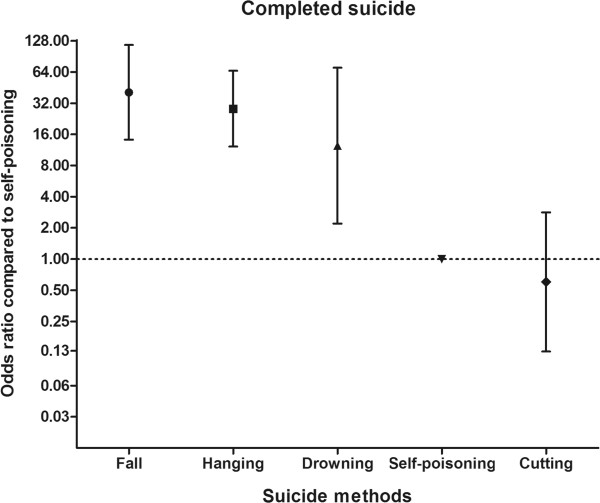
**Odds ratio for death prior to discharge by each suicide method.** Each plot represents the odds ratio and 95% confidence interval compared with self-poisoning.

Finally, in the high lethality methods group, 39 patients (43.8%) died prior to hospital discharge. In the low lethality methods group, only 11 patients (2.3%) died. Compared with patients treated for self-poisoning, the ORs for completed suicides were 40.73 (95% CI = 14.22 to 116.67), 28.28 (95% CI = 12.19 to 65.62), 12.44 (95% CI = 2.20 to 70.32), and 0.60 (95% CI = 0.13 to 2.83) for patients treated for falling, hanging, drowning, and cutting or piercing, respectively (Figure 2).

**Table 2:** **Multivariate logistic regression analysis for factors associated with the use of high lethality methods for attempted suicides**

Variable	Odds ratio for critical methods	95% Confidence interval	p
Age	1.02*	1.01 – 1.03	< .01
Previous high lethality methods	7.22	3.06 – 17.04	< .01
Alcohol coingestion	0.59	0.35 – 0.99	.04

*: Odds ratio per year.

## Discussion

The present study analyzed the sociodemographic profiles of patients who attempted suicide using high lethality methods relative to those who used low lethality methods of suicide. The overall picture that emerged was that age and previous attempted suicide using high lethality methods were independent predictors of attempted suicide using high lethality methods other than self-poisoning or cutting. On the other hand, alcohol co-ingestion before an attempted suicide was significantly associated with using a low lethality method.

Attempted suicides, especially repeated attempts, are high-risk factors for subsequent suicide [11–13]. In the year following an attempted suicide, the average rate of repeated, non-fatal attempts was reported to be 17% [8]. In follow-up studies conducted 5 to 37 years after an attempted suicide, the risk of suicide was reported to be 3 to 13% [13–18]. The risk for suicide among self-harming patients is estimated to be 40 times higher than among the general population [19, 20]. But, it is unclear how to identify individuals who would be at risk of subsequent suicide among suicide attempters. Of the method of attempted suicides, the high lethality methods category included hanging, drowning, the use of firearms or explosives, falling or jumping from a great height, and gassing. These methods are less commonly used, but they bear a higher mortality risk. In our study, these methods accounted for only 15.9% of total attempted suicides but for 78.0% of total deaths prior to discharge. Therefore, assessment of risk factors for choosing high lethality methods compared with other suicide methods may help in designing suicide prevention strategies.

There have been some recent attempts to uncover risk factors for hanging compared with other suicide methods. One study compared hanging suicides with all other suicides in Lithuania and reported that hanging victims were more likely to be male and rural-dwelling than other suicide victims [21]. These victims were also older and had lower education levels. In our study, patients who had made a previous attempt using a high lethality method were 7.8 times more likely to use a high lethality method again. In particular, the method used during an attempted suicide predicts a later completed suicide after adjustment for sociodemographic variables and psychiatric disorders. Therefore, intensified aftercare is warranted following attempted suicides involving high lethality methods [7]. Age was also associated with the use of high lethality methods. This is in line with the previous study that showed high risk and low rescue scores in elderly suicide attempters [22]. It is well known that acute alcohol use was associated with both attempted suicides and completions [23, 24]. Cherpitel et al. reviewed studies that included data on acute alcohol use in completed suicides and attempted suicides [24]. However, a wide range of alcohol positive cases was found: 10 to 69% for suicide and 10 to 73% for attempts. The mean percentage of alcohol use was 37% and 40% for suicide and attempt, respectively. These highly variable and uncontrolled results make it difficult to draw conclusions about the relationship between acute alcohol use and suicidal behaviors. According to our results, acute alcohol use before an attempted suicide was an independent predictor of choice of low lethality method.

Some of our findings are inconsistent with those published in the literature. Men who attempt suicide are known to engage in more dangerous methods, often resulting in severe life-threatening consequences [25, 26]. However, in contrast with previous studies, men did not use more high lethality methods in the present study. In one study regarding of annual changes in the pattern of suicide methods in Korea, the United States, and Finland, the most common suicide methods were firearm in American males and hanging in Finland male. On the other hand, self-poisoning was most common suicide method among females in both countries. Interestingly, in Korea, hanging has been the most common method of suicide among both males and females [27].

Mental disorders are predictive of suicidal behavior [28]. According to a recent meta-analysis, 4.9% of patients with schizophrenia die of suicide, most of them soon after symptom onset [29]. Additionally, those with schizophrenia tend to use highly lethal suicide methods [30]. According to the results of this study, individuals with underlying psychotic disorders are more likely than other individuals to use high lethality methods and are 2.2-fold higher. In China, a national case–control psychological autopsy study reported an overall rate of mental disorders among completed suicides of 63%, which is much lower than the rate of 90% reported in psychological autopsy studies from other countries [31]. Recent study revealed that demographic and socio-cultural risk factors for suicide in Asia are different from Western countries [28, 31, 32]. In our study, psychiatric disorders were present in 41.6% of patients in the high lethality methods group and in 39.1% of patients in the low lethality methods group. Finally, schizophrenia was not associated with high lethality suicide methods. We believe that suicide is less associated with mental illness also in Korea, and discrepancy between the present study and previous studies may come from Korea’s suicide feature.

This retrospective, single-center study has several limitations. First, we only included patients who came to the hospital to receive treatment. For this reason, some completed suicides, especially using high lethality method, may not be included in the present study. Second, the present study did not examine the patients’ attempted suicide motives, social class, marital status, or other medical illnesses. Third, the sample is relatively small and from a single hospital. For these reasons, our results should be interpreted cautiously.

## Conclusions

This retrospective chart review suggests that patients who are older and have had previous attempted suicide using high lethality methods are more likely to use high lethality methods in future attempts and are more likely to have completed suicides. On the other hand, acute alcohol use before an attempted suicide was an independent predictor of choice of low lethality method.
